# Dimorphic evaluation of hippocampal changes in rat model of demyelination: A comparative functional, morphometric, and histological study

**DOI:** 10.1002/brb3.2723

**Published:** 2022-07-21

**Authors:** Aref Nooraei, Kaveh Khazaeel, Marzieh Darvishi, Zohreh Ghotbeddin, Zahra Basir

**Affiliations:** ^1^ Faculty of Veterinary Medicine Department of Basic Sciences Shahid Chamran University of Ahvaz Iran; ^2^ Stem Cell and Transgenic Technology Research Center Shahid Chamran University of Ahvaz Ahvaz Iran; ^3^ Faculty of Medicine Department of Anatomy Ilam University of Medical Sciences Ilam Iran; ^4^ Biotechnology and Medicinal Plants Research Center Ilam University of Medical Sciences Ilam Iran

**Keywords:** demyelination models, hippocampus, inflammation, morphometric study

## Abstract

**Background:**

Multiple sclerosis (MS) is the most common autoimmune disease. Progressive depletion of the brain and spinal cord tissue appears at the onset of the disease. Several studies have shown the increased size of the ventricles of the brain and decreases in the area of the corpus callosum and the width of the brain. Other important symptoms of this disease are cognitive, learning, and memory disorders.

**Aim:**

The aim of this study was to compare morphometric, histological, and functional changes in the demyelination model in both sexes.

**Materials and methods:**

In this experimental study, male and female Wistar rats were studied in four experimental groups. Demyelination was induced by the injection of ethidium bromide in the ventricular region. The chronic effect of demyelination on spatial memory, movement, and coordination was investigated using the Morris Water Maze (MWM), and clinical and balance beam tests, respectively. Myelin degradation, cell death and neurogenesis were estimated using Luxol Fast Blue staining and immunohistochemistry (Caspase‐3 and Nestin markers). In addition, morphometric findings were recorded for the brain and hippocampus (weight, volume, length, width).

**Result:**

Demyelination increased the time and distance index and decreased the residence time in the target quarter in the water maze test (*p* < .001). It also increases the neuromuscular and modified neurological severity score (*p* < .01). Demyelination increases caspase‐3 (*p* < .05) expression and decreases Nestin expression (*p* < .001), which are directly related to the extent of damage.

**Conclusion:**

This study showed an interaction between hippocampal structural and functional networks in explaining spatial learning and memory in the early stages of MS.

## 1 INTRODUCTION

Multiple sclerosis (MS) is an autoimmune disease of the central nervous system (CNS) characterized by inflammation, demyelination, axonal damage, plaques, astrogliosis, and microglial activation (Chu et al., 2021; Libbey & Fujinami, 2021; Tambalo et al., 2015). In recent years, research has confirmed that MS is controlled by T‐cell‐induced autoimmune reaction, which leads to myelin destruction (Koike & Katsuno, 2021; Monaco et al., 2020). Demyelination has various deleterious effects on a different parts of brain regions, such as white matter (corpus callosum) and gray matter (cortex, hippocampus, and cerebellum) (Bauer et al., 2020; Cohan et al., 2021). The hippocampus is an active region of the brain where neural stem cells continue to proliferate and differentiate into neuroblasts and granule cells throughout life (Jurkowski et al., 2020; Nickell et al., 2020). This part of the brain is especially involved in memory and learning processes and in the consolidation of long‐term memory from short‐term memory, which is instigated by neurogenesis (Avigan et al., 2020; Chu et al., 2021). Several studies have confirmed the presence of plaques in the hippocampus in MS, which can be a cause of cognitive deficits and neuropsychological abnormalities (Berdugo‐Vega et al., 2020; Dobrynina et al., 2020). Findings indicate that steroid hormones may be involved in the pathophysiology of MS (Ziehn et al., 2012). However, the present study aimed to evaluate the dimorphic changes of the hippocampus in the demyelination model of rats without the intervention of steroid hormones on function, morphometry, and histology. To identify anatomical and physiological factors associated with MS, several animal models, including toxin‐induced models, have been established (Bebo et al., 2001; Ziehn et al., 2012). Direct injection of ethidium bromide (EB) into the lateral ventricles as a chromatin‐disrupting agent has been used for the induction of neural cell degeneration and to study the biology of demyelination (Blakemore, 1982; Mazzanti et al., 2007). EB induces cytotoxic activities and the production of free radicals, thus causing the oxidative stress that is the most common complication of MS (Goudarzvand et al., 2016; Woodruff & Franklin, 1988). Decreased brain volume is one of the most important aspects of MS. Brain atrophy is affected by several factors, including apoptosis with oligodendrocyte depletion, increases in demyelination, loss of astrocytic volume, and Wallerian degeneration, which contribute to focal lesions in white matter and gray matter (Paxinos & Watson, 1982; Sim et al., 2000; Ubogu et al., 2012; Wegner et al., 2006; Zivadinov & Bakshi, 2004). Since no studies on macroscopic and microscopic examination of the brain in demyelinated rats in the form of dimorphism have been performed. Therefore, the present study aimed to evaluate the effects of intraventricular injection of ethidium bromide as a focal model for evaluating morphometric and behavioral changes in the hippocampus in rats of both sexes. Thus, the present study aimed to assess the effects of intraventricular injection of ethidium bromide as a focal model to evaluate morphometric and behavioral changes in the hippocampus in rats of both sexes.

## 2 MATERIAL AND METHODS

### 2.1 Animal grouping

Experiments were done according to the Ethical Committee (by Ethical code: EE/99.3.02.55591/scu.ac.ir) for Animal Care Use of Laboratory Animals at the Shahid Chamran University of Ahvaz, Ahvaz, Iran. Male and female Wistar rats (M: 300–400 g and F: 250–300 g) were used. Animals were housed in a controlled environmental condition with 12‐h light/dark cycles with standard rat food and water ad libitum. Vaginal smears from all female rats at the beginning of the work and also after the onset of MS symptoms in rats, to confirm that female rats were in the same sex period. So the estrous cycle did not affect results (Ajayi & Akhigbe, 2020). Forty adult rats of both sexes (20 each) were divided randomly into four groups. These four groups (*n* = 10 per group) were formed as follows: the MN group (10 male rats with normal diet and normal saline injection) and the FN group (10 female rats with normal diet and normal saline injection), the MEB group (10 male rats with normal diet and ethidium bromide (EB) injection), and the FEB group (10 female rats with normal diet and ethidium bromide [EB] injection).

### 2.2 Surgical procedures

Surgery was performed under anesthesia (ketamine [50 mg/kg] and xylazine [10 mg/kg]: Alfasan Company, Woerden, the Netherlands) and aseptic conditions. Rats were placed on the stereotaxic instrument in the skull‐flat position. Demyelination was induced unilaterally by a direct single injection of 3 μl of 0.01% ethidium bromide (Sigma, Germany) in sterile 0.9% saline (Ajayi & Akhigbe, 2020) at the rate of 1 μl/min into the intracerebral ventricle (ICV) by stereotaxic devices (AP = −2.8; ML = +1.8; DV = +2.5). It was given that trypan blue dye was used in these rats for injection to determine the best point. These samples were fixed in 10% formaline solution after injection. One of the advantages of this color combination is that it does not disappear in stabilizing solutions and is visible (Dai et al., 2011).

### 2.3 The functional assessments

#### 2.3.1 Neuromuscular severity scores

Neuromuscular severity scores (NSS) were performed to assess locomotive activity. The NSS test measures the fore and hind limb weakness of the rat and its body position. The clinical signs of weakness were recorded using a 6‐point scale where 0 = normal strength and tone without any symptoms, 1 = reduction of tail tone with mild weakness, 2 = weakness of one hind limb (moderate weakness), 3 = paralysis of both hind limbs (severe weakness), 4 = paralysis of both fore limbs, 5 = premorbid state, 6 = death. Scoring was done once before surgery, and then, following the operation, scoring was conducted once a week for 12 weeks by two independent examiners (Sohaili et al., 2020).

#### 2.3.2 Modified Neurological Severity Scale (MNSS)

The ability of the animals to keep their balance was assessed using a balance beam test that is part of the Modified Neurological Severity Scale (MNSS). MNSS consists of a 6‐point scale (normal = 0; maximum = 6) as shown in Table 1.

**TABLE 1:** The Modified Neurological Severity Scale (MNSS)

Test	Score
Posture balances	**0**
Catches of beam	**1**
One limb falls down from the beam and hugs the beam	**2**
Two limbs fall down from the beam and hug the beam in more than 60 s	**3**
Attempts to balance but falls down in more than 40 s	**4**
Attempts to balance but falls down in more than 20 s	**5**
No attempt to balance and falls down from the beam	**6**

### 2.4 Footprint test

**FIGURE 1:**
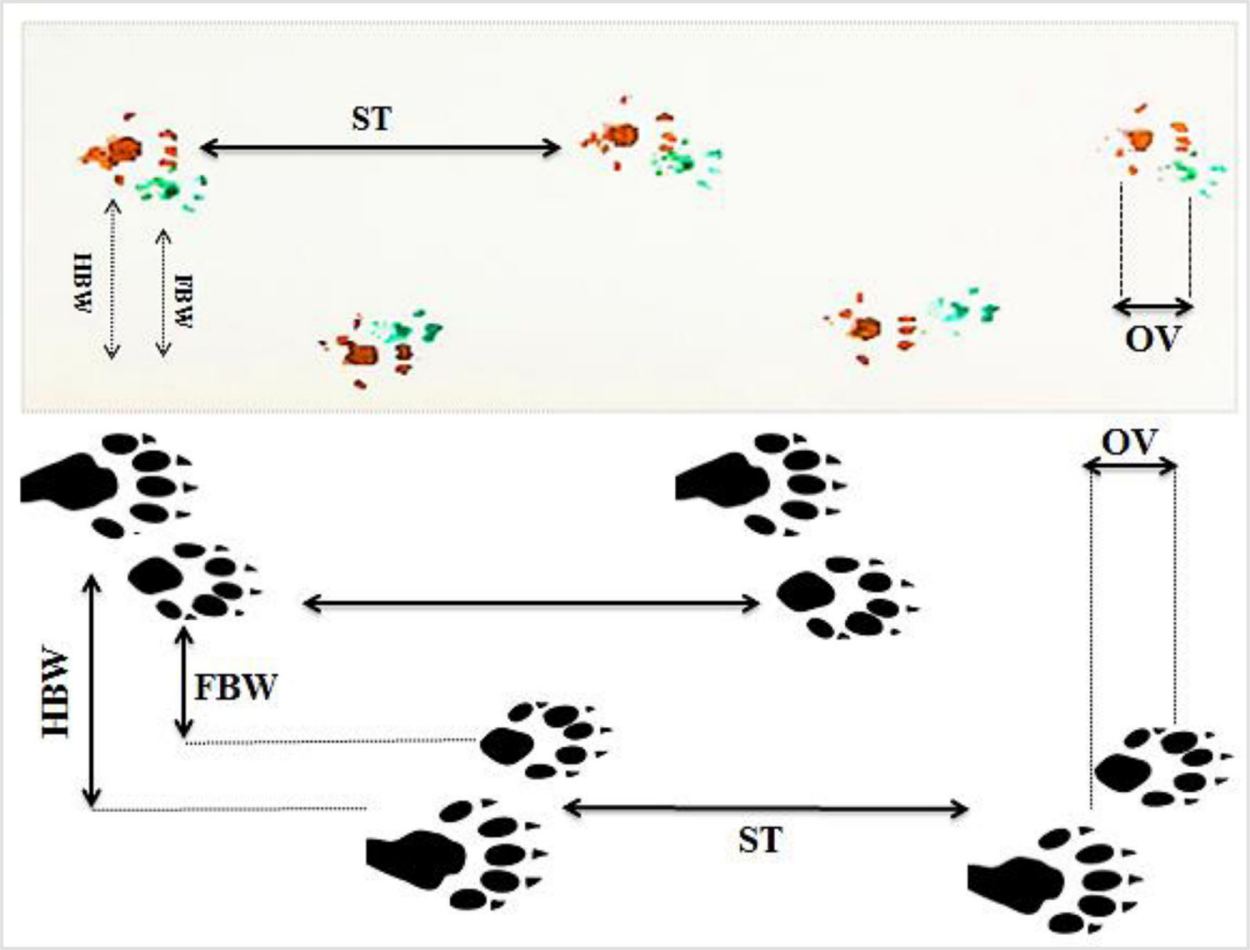
Footprint parameters (stride length (ST), hind‐base width (HBW), front‐base width (FBW), overlap of front, and hind footprint (OV)

To obtain the footprints, animals were habituated one day before starting experimental tests. For testing, the rat forelimb and hind limb were pressed on green and red non‐toxic paints. Then animals were allowed to walk across a sheet of paper through an open field (a runway that was 100 cm long, and 8.5 cm wide illuminated by a light) leading to a dark box. Footprint patterns were evaluated for four step parameters as indicated in Table 2 (Figure 1). After that, for each step parameter, four values were measured from each run and finally the average of each set was analyzed (Table 2) (Sohaili et al., 2020).

**TABLE 2:** The footprints test

Index	Test
Stride length (ST)	The average distance between each stride
Hind‐base width (HBW)	The average distance between left and right hind footprints
Front‐base width (FBW)	The average distance between left and right front footprints
Overlap of front/hind footprint (OV)	Distance from front footprint/hind footprint overlap

### 2.5 Morris water maze procedure

To assess spatial memory and learning we used the Morris Water Maze (MWM) procedure. Vorhees and Williams (2006) have explained MWM extensively. A tracking system was placed above the maze to analyze the distance traveled, time spent to reach the platform, and time spent in the target zone. For recording sensory motor function, swimming speed was investigated. The tests were performed in a pool made of black fiberglass. This pool is 1.2 m in diameter with a height of 0.8 m filled with water at 25°C. The target platform with a diameter of 10 cm was submerged 1−1.5 cm under the surface of the water. Start points for the rats were defined on the outside of the pool as north (N), south (S), east (E) and west (W). The protocol consisted of five training days: the first 4 days with a visible platform and day 5, which allows the animal to swim to the submerged platform placed in the tank.

### 2.6 Brain and hippocampal extraction and gross morphological assessment

Twenty rats consisting of five males from each MN and MEB groups and five females each from the FN and FEB groups were utilized to study gross morphometrics of the rat hippocampus and brain. For morphological assessment, rats were anesthetized with intraperitoneal injection of ketamine‐xylazine (ketamine 50 mg/kg) and (xylazine 10 mg/kg) and then perfused with 4% paraformaldehyde in phosphate‐buffered saline. Each skull was exposed after skinning and stripping off and then craniotomy was performed. The falx cerebri and tentorium cerebelli were both pulled. Brain and hippocampus were removed and fixed in 4% paraformaldehyde. The different components of the brain were analyzed with the naked eye. These included weight, volume, length, width and thickness of the brain as well as the volume and length of the hippocampus from the ventral to the dorsal, and finally the ratio of the volume of the hippocampus to the brain. Investigation of gross anatomical structures was determined referring to standard documentation on rodent anatomy (Aliheydari et al., 2020).

### 2.7 Histological assessment of hippocampus

In order to assess histological changes following demyelination, Nissl staining was used. Briefly, 12 weeks after the EB injection, rats were anesthetized and perfused with 4% paraformaldehyde in phosphate‐buffered saline. The brains were removed and fixed for three days. Then the brain samples were embedded in paraffin. Coronal sections were supplied by a microtome at 7 μm thickness, then stained using 0.1% Cresyl Violet for 10 min and 0.1 % Luxol Fast Blue (LFB) solution at 60°C for 12 h. Sections were visualized using an upright microscope (kern. German). Intact neurons were marked as neurons with low basophilic neurons and Nissl bodies, while dark neurons with hyperbasophilic cytoplasm and shrunken morphology were characterized as apoptotic neurons. For immunohistochemistry staining, sections were permeated with triton x‐100 (0.3%) blocked in goat serum 10%. Samples were treated overnight with primary antibodies, including mouse anti‐Caspase3 monoclonal antibody (1:500; Abcam, Cambridge, UK) and mouse anti‐nestin monoclonal antibody (1:500; Abcam). After 24 h these were washed in PBS and incubated with a secondary antibody (rabbit anti‐mouse antibody conjugated with FITC) for 45 min at room temperature. Nuclei were stained with propidium iodide (PI, Sigma, 1:10,000) for 3 min. Cross‐sections were evaluated under a fluorescent microscope at 200× magnification (kern. German). The caspase‐3 and Nestin immunoreactive areas were randomly selected, and the number of positive cells in 5 preselected areas from each of 3 consecutive slices in all animals were counted.

### 2.8 Statistical analysis

Statistical analysis of the data obtained from physiological tests and body weight were evaluated by repeated measures analysis of variance. Macroscopic and microscopic parameters were evaluated by two‐way analysis of variance and supplementary Tukey's test, *p* ≤ .05 was considered a significant difference.

## 3 RESULT

The mean weight of the rats in the MN and FN groups was compared to rats that received EB (MEB and FEB groups) in each week. The weight of rats in the MEB and the FEB group was significantly different between the 4th and 12th weeks and there were significant differences between the intact groups (MN and FN) and the injury groups (MEB and FEB). The weight of rats in the 12th week in the injury groups after EB injection showed a small decrease (Table 3).

**TABLE 3:** Mean ± SEM of weight in the different experimental groups

Groups	Preoperation	4 weeks	8 weeks	12 weeks
FN	230.3 ± 8.08	227.4 ± 6.9	227.1 ± 4.9	229.8 ± 5.7
MN	324.1 ± 4.53	317.7 ± 1.9	321.5 ± 7.06	322.2 ± 2.7
FEB	234.2 ± 8.5	233.6 ± 8.5	229.2 ± 8.8	223.5 ± 8.2**
MEB	314.7 ± 3	295.6 ± 6.2	290.7 ± 6.3	284 ± 6.8***

**p < .01 and ***p < .001 indicate difference in weight between the 1st and 12th weeks.

### 3.1 Clinical assessment

**FIGURE 2:**
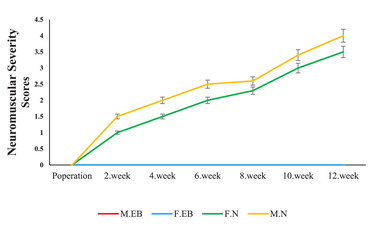
Scores of neuromuscular severity from the MN group (intact male rats with normal saline injection), FN group (intact female rats with normal saline injection), MEB group (male rats with ethidium bromide injection), and FEB group (female rats with ethidium bromide injection)

Rats in the demyelination groups (MEB and FEB) showed moderate weakness that increased to severe hind‐ and forelimb weakness. This score gradually increased until the end of the 12th week and was accompanied by weight loss. Compared to the intact groups (MN and FN groups), this difference was statistically significant. In addition, comparing the data between the two sexes showed that the male group (MEB) had a higher score than the female group (FEB) from the 1st to the 12th week and this difference was significant. In the MN and FN groups, the results are seen as a straight line at the baseline (Figure 2).

### 3.2 Balance beam test

**FIGURE 3:**
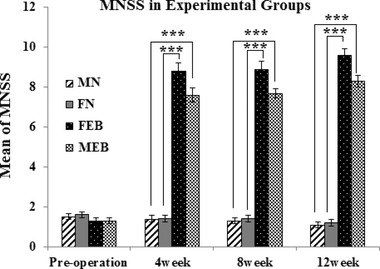
Balance was measured using MNSS test (balance beam test) in male and female saline groups, male and female EB groups(0 = normal and 6 = maximum). Values are expressed as mean ± SEM (*p < .05, **p < .01, and ***p < .001), based on repeated measure ANOVA

Animals were tested to evaluate balance and motor coordination using the Balance Beam Test. All movement errors of the animal were recorded, including errors per posture balances, hugs of the beam, and one or two limbs falling from the beam (Figure 3). Analysis shown that injury groups of male (MEB) and female (FEB) rats had significantly higher scores compared with the intact groups (MN and FN) at 12 weeks (*p *< .001). In addition, the FEB group had significantly lower scores compared with the MEB group (*p* < .01).

### 3.3 Footprint test

**FIGURE 4:**
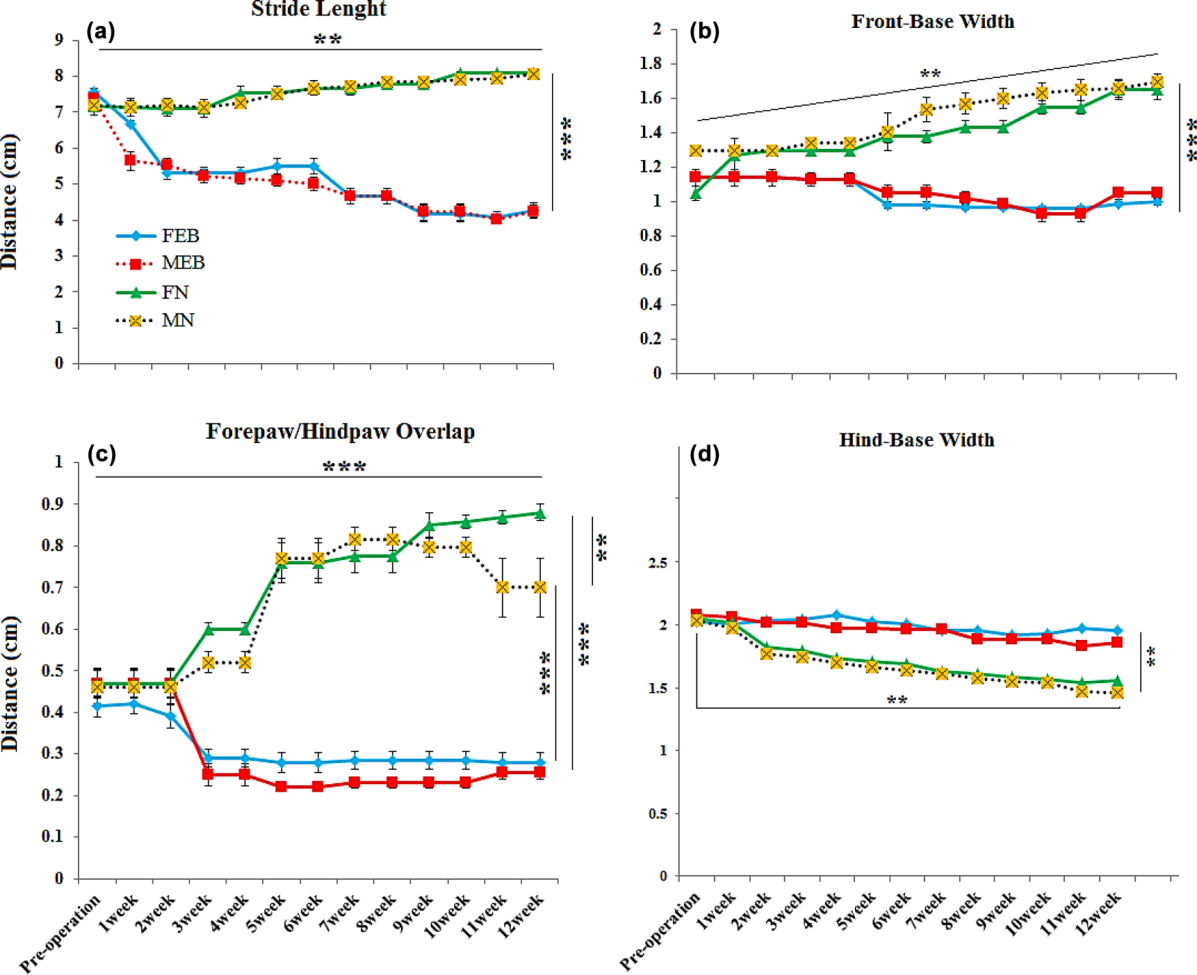
Footprint patterns in experimental groups that include, ST: stride length (a), FBW: front‐base width (b), OV: distance between front and hind footprint overlap (c) and HBW: hind‐base width (d). Values show means ± SEM by rats of each group at 1–12 weeks (**p < .01; ***p < .001; with Bonferonni's correction), based on repeated measure ANOVA

We analyzed footprint parameters for 12 weeks to assess the locomotor activity of the errors per steps. Footprint patterns of the injury groups (MEB and FEB) and intact rats (MN and FN groups) from the 1st to the 12th week following the experimental procedure are presented in Figure 4. At all weeks of the experiment, the MN and FN groups walked in a straight line with regular steps. In contrast, the MEB and FEB groups walked from side to side, with unregularly steps, a shorter gait, and confounded movements. In the MN and FN groups, a quantitative type of gaiting with four parameters was assessed: ST, FBW, HBW, and OV. The ST of the healthy groups (MN and FN) was a straight line and was statistically significantly higher than the injured groups (*p* < .001) (Figure 4a). Comparison of the FBW index between the healthy (MN and FN) and injured groups (MEB and FEB groups) showed that this index was higher in the MEB and FEB groups and this difference was also statistically significant (*p* < .001). In addition, the increase in this index continued from the first to the 12th week and was statistically significant (*p* < .01) (Figure 4b). However, the ST and FBW indexes did not show significant differences between the two sexes. OV indicated a uniformity of step periodicity in the healthy groups (MN and FN) from weeks 1 to 12 and illustrated a lesser overlap between front and hind paws compared with the MEB and FEB groups (*p* < .001) (Figure 4c). In comparing the two sexes, no significant difference was observed in the movement and overlap of front and hind paws. The overlap of paws in the MEB and FEB groups increased from the 1st to the 12th week and confirms this irregular and chaotic movement; in comparison between the two sexes, the MEB group showed a significantly greater increase in the OV index than the FEB group (*p* < .01). Examination of the HBW index also showed that in the injury group, it was lower than in the healthy group and that this decrease was seen from the 1st to the 12th week, but there was no significant difference between the sexes (Figure 4d).

### 3.4 Morris water maze

**FIGURE 5:**
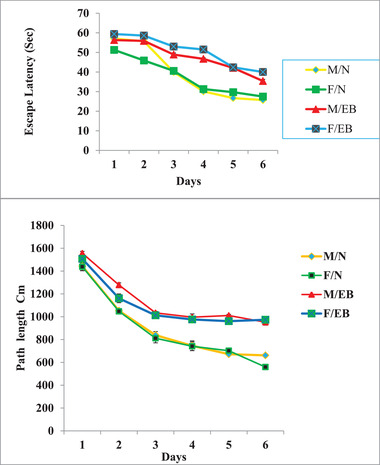
Morris water maze test results during 6 days of training between healthy (MN and FN) and injured groups (MEB and FEB) (**p < .01), based on repeated measure ANOVA

In Figure 5, according to the analysis of time data, the average time spent to reach the platform (Escape Latency) in the healthy male and female groups from the second day to the sixth day showed a significant decrease. In the demyelination groups (MEB and FEB), this decrease was also observed from the second to the sixth day and a comparison between the two sexes showed that the male group spent less time reaching the platform than the female group; this difference was statistically significant (*p* < .01) (Figure 5a). In linear diagram (Figure 5b), according to the analysis of distance data, the average distance traveled to reach the platform (path length) in the healthy male and female groups from the first to the sixth day showed a significant decrease compared with the injury group (*p* < .01). In comparison between the injury groups, the study did not show a significant difference between the two sexes.

### 3.5 Gross morphological assessment of the hippocampus

**FIGURE 6:**
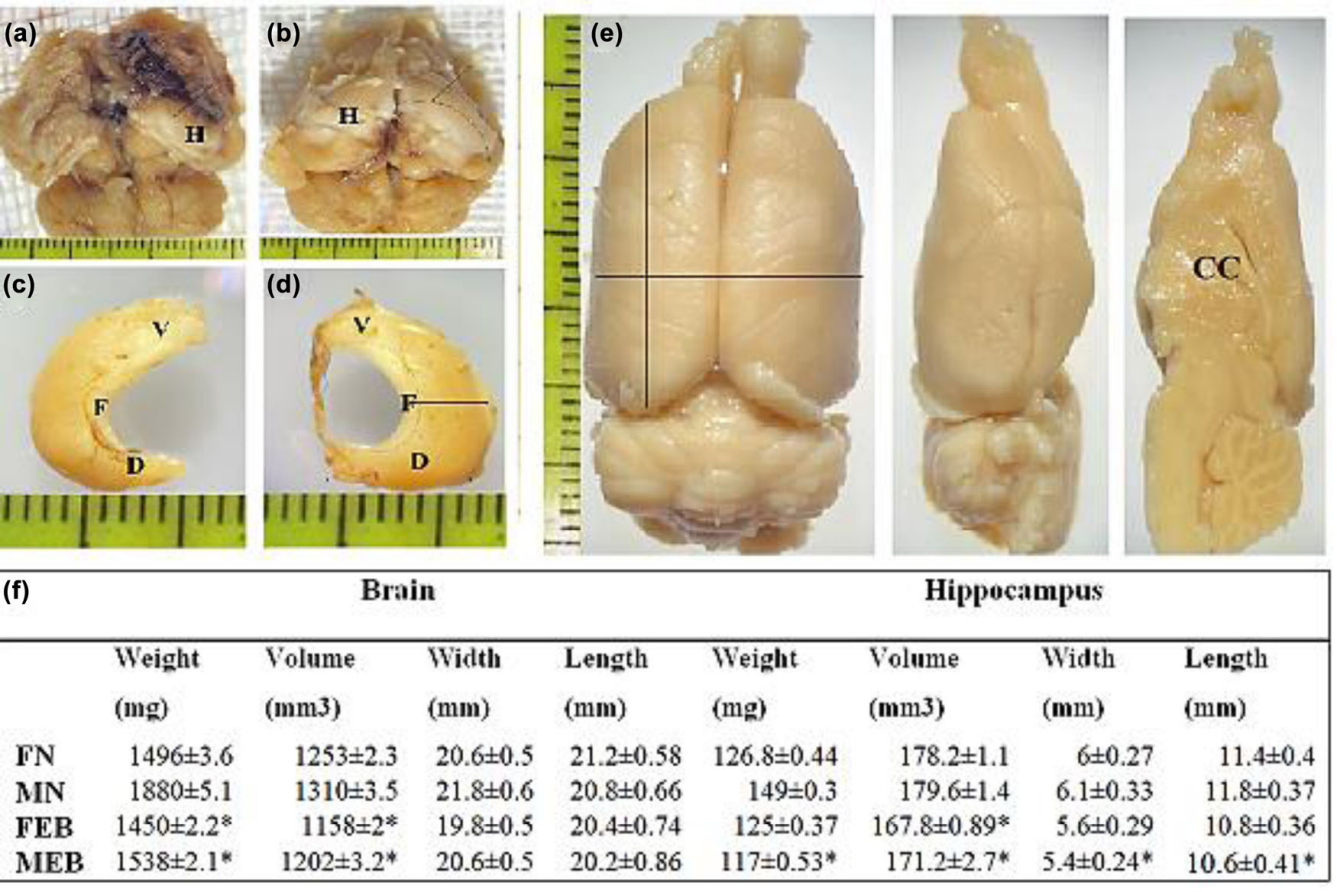
Dorsal view of the brain. H: hippocampus, V: ventral, D: dorsal, F: fimbria, CC: corpus callosum. Values indicate means ± SEM by rats of each group at 12th week (weight, volume, length, and width brain and hippocampus)

Figure 6 shows the findings of macroscopic examination of the brain and the hippocampus. Brain weights in the MN, FN, MEB, and FEB groups were 1880 ± 5.1, 1496 ± 3.6, 1538 ± 2.1, and 1450 ± 2.2 mg, respectively. Comparison of the results between the male and female groups showed that brain weights in the male group were higher than in the female groups but not statistically significantly higher. In addition, brain volume in the MN, FN, MEB, and FEB test groups were 1253 ± 2.3, 1310 ± 3.5, 1202 ± 3.2, and 1158 ± 2 mm^3^, respectively. Comparison between the healthy and injured groups showed that the brain weight in the injured groups was less than in the healthy groups but was not statistically significant.

### 3.6 Histological assessment

**FIGURE 7:**
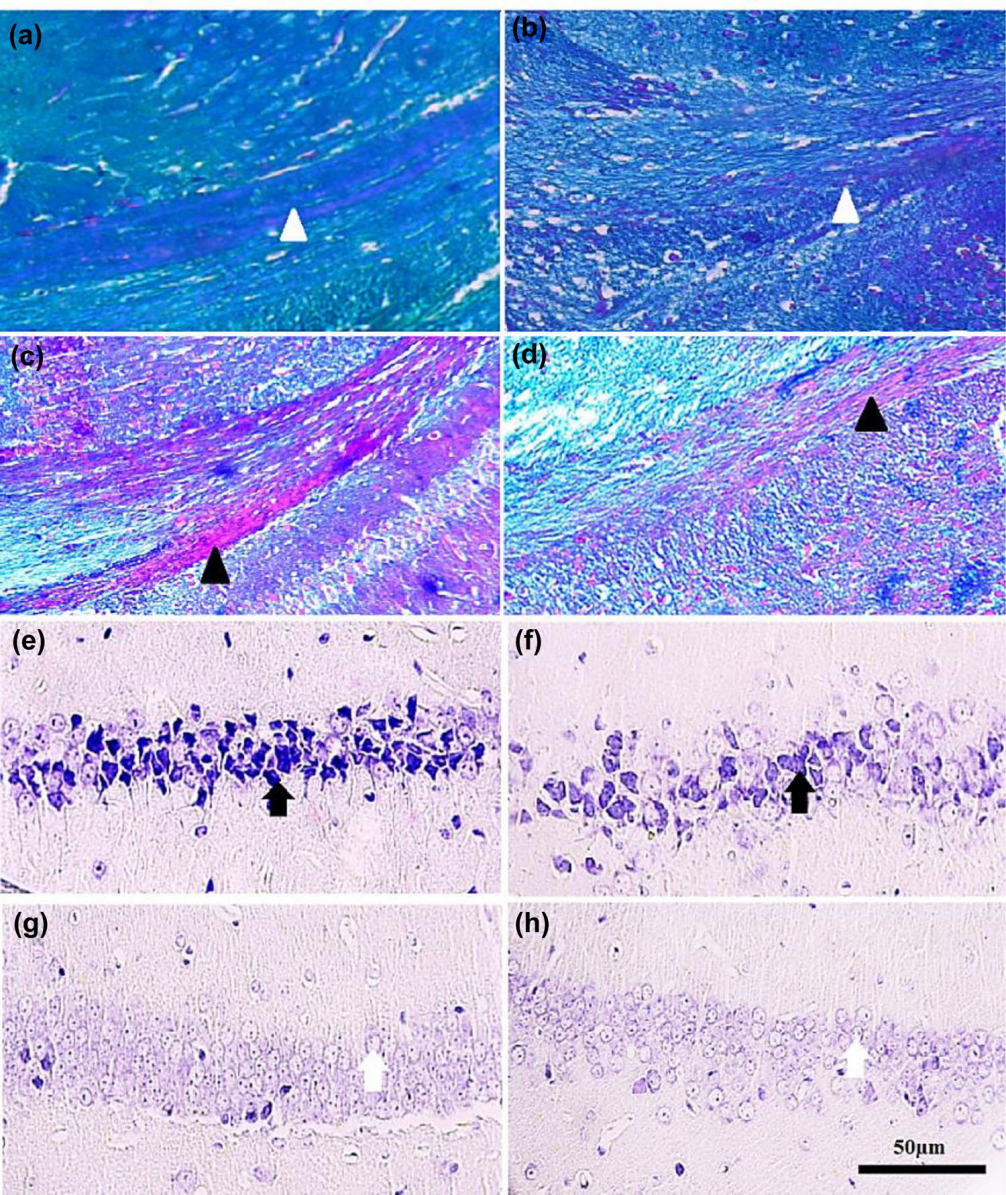
(a–d) Effect of EB on myelin sheath in demyelination model of rat. The blue areas show intact regions whereas the pink areas indicate demyelinating regions. The white arrowhead is the myelin sheath and intact area and the black arrowhead is a demyelination region. (e) and (f) indicate the CA1 region of the hippocampus and the arrow shows a dark cells or apoptotic cells

Figure 7a and 7b shows tissue sections of the corpus callosum that were taken from healthy specimens staining myelin axons with LFB (MN and FN, respectively), whereas Figure 7c and 7d shows the male and female demyelination groups (MEB and FEB respectively). Injury groups show myelin degradation and are observed in red color. Using Cresyl violet staining, dark cells can be seen in large numbers in the affected groups of both sexes in the CA1 region of the hippocampus, while in healthy groups, these cells are seen in small numbers.

**FIGURE 8:**
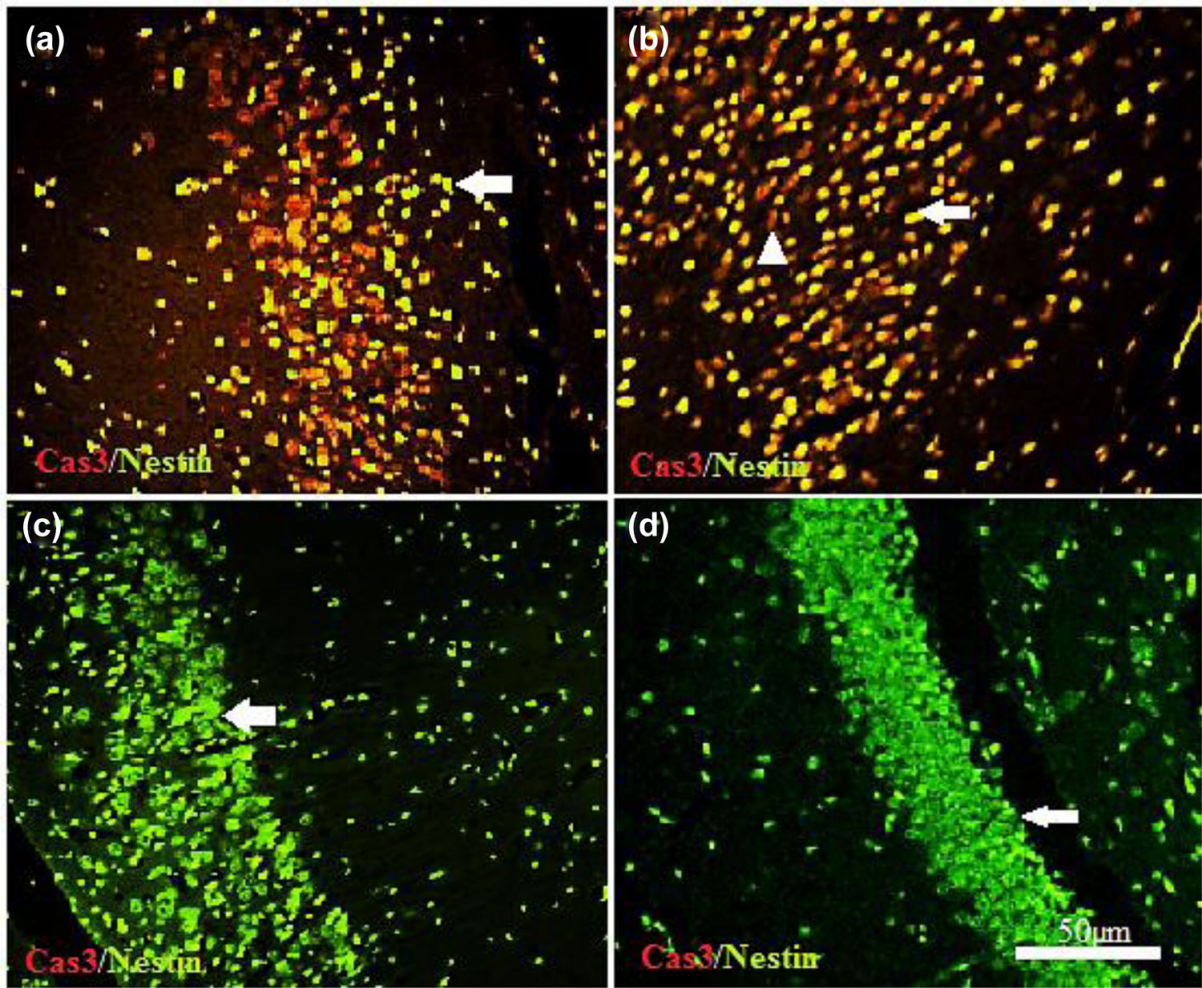
The expression of two markers Nestin and caspase 3 in the C1 region of the hippocampus. The white arrowhead indicates the caspase 3‐positive cell and the arrow show Nestin‐positive cell

Figure 8 shows the expression of two markers: Nestin (neurogenesis marker) and caspase‐3 (apoptotic marker) in the C1 region of the hippocampus. Using immunohistochemistry and double staining technique, two Nestin antibodies (green) and caspase‐3 (red) were examined. The results show that in the healthy male MN (c) and female FN (d) groups, the expression of Nestin was high and the expression of caspase‐3 was low, which was confirmed by fluorescent cell count. However, in the male MEB (a) and female FEB (b) injury groups, the opposite was observed.

## 4 DISCUSSION

The prevalence of MS in women is almost twice that of men, which is not yet a definite reason for this difference. To date, no morphometric and behavioral studies between the sexes have been performed in relation to MS. Cognitive disorders such as memory impairment and hippocampal demyelination are common in MS patients (He et al., 2002). There is widespread agreement that memory and behavior depend on hippocampal health, with numerous studies showing that damage to the hippocampus leads to memory and behavioral disorders (Bosoko & Langdon, 2020). Autoimmune encephalomyelitis is the most widely used demyelination model (Constantinescu et al., 2011). Numerous techniques have been proposed to create models of brain demyelination, many of which are not suitable for examining cognitive activities such as memory and learning due to movement disorders. Ethidium bromide (EB) is a toxic drug that induces demyelination (Bannerman et al., 2005; Linker & Lee, 2009). In the present study, EB caused demyelination and axonal destruction accompanied by impaired coordination and balance such that this finding is in agreement with data obtained from other researchers (De Stefano et al., 2014). In a study conducted by Sohaili et al. In 2020, the effect of valproic acid on demyelination was studied. In this study, footprint test was used to assess the balance and movement of mice. This is followed by movement disorders, imbalance, visual impairment, and muscle weakness caused by this disease which is consistent with the present study (Sohaili et al., 2020). These results show that induction of demyelination with EB causes morphological, histological, and behavioral symptoms akin to MS in rats. Morphometric investigation of brain volume and weight showed that EB demyelination can lead to a decrease in total brain volume and weight, and this decrease was observed in both sexes, despite numerous findings that the probability of occurrence and severity of symptoms in females is often higher than in males. Our findings showed, that the total brain changes in males were greater than in the female rats. Despite the strong female bias in MS incidence, culminating evidence from natural history studies, and imaging and pathology studies suggest that males who develop MS may exhibit a more rapid decline in disability and cognitive functioning than women, insights provided by imaging and pathology studies in MS, and in MS animal models have suggested that axons or myelin may be more vulnerable to autoimmune attacks in males, which is in line with the results of the present study (Dunn et al., 2015). Decreases in volume, weight, length, and width were observed in the EB model. Several human studies using magnetic resonance imaging findings have shown reduced brain volume in the ventricles, white matter, and gray matter of the brain in MS patients (Dunn et al., 2015; Horakova et al., 2009). In this study, a decrease in brain volume was associated with a decrease in the volume and weight of the hippocampus, which is directly related to histological findings. In the affected group where a decrease in hippocampal volume was observed, impairment in behavioral data, including movement, balance, and spatial memory, was also observed. In the studies of Ghaffari et al. (2013), they used the glutotoxic substance ethidium bromide to cause MS and the Morris water maze test to assess spatial memory. The mean time to reach the platform in the healthy saline and male and female groups from the second to the sixth day shows a significant decrease. In the affected groups, this decrease was observed from the second to the sixth day and the comparison between the two sexes showed that the male group spends less time than the female to reach the platform and this difference is statistically significant. This effect was more pronounced in male rats than in females (Ghaffari et al., 2013). The present study indicates that induction of MS by EB in males and females led to a deterioration in short‐term memory. This data is in agreement with data obtained from other researchers (Juliette et al., 2021; Strik et al., 2021; Yao et al., 2020). One of the main features of MS animal models is apoptotic neuronal cell death (Artemiadis & Anagnostouli, 2010; Thornton & Raz, 1997). The morphological hallmarks of apoptosis include chromatin condensation with a dark view, cell shrinkage, and the formation of apoptotic bodies. Caspase‐3 is activated in the early stages of apoptosis. In the present study, the expression of caspase‐3 (apoptotic marker) in the hippocampus of the demyelination groups was more pronounced, indicating cell death and subsequent reduction in hippocampal volume, which is in agreement with the findings of other researchers (Chen et al., 2015; D'Amelio et al., 2010; Huang et al., 2018). Jin et al. (2014) showed that EB injection and induction of demyelination led to increased expression of caspase‐3 and increased numbers of TUNEL‐positive cells in the hippocampal dentate gyrus. In addition, our findings showed the expression of Nestin in the injury group was lower, which indicates a decrease in the expression of brain factors and less able to repair the area.

## 5 CONCLUSION

In conclusion, our study showed an interaction between hippocampal‐related structural and functional networks in explaining spatial learning and memory in the early stages of MS model. By inducing demyelination in the brain and hippocampus, spatial learning and memory functions, as well as coordination of movement and balance, were reduced, while functional organization seemed to be able to maintain spatial memory through strengthening short‐term communication. On the other hand, it seems that this method of inducing a demyelination model can depict morphological, histological, and behavioral injuries without complete movement destruction. Therefore, this method of injecting EB into the lateral ventricles will provide a suitable model for future studies by researchers. The final results of this study showed that although the prevalence of MS in females is higher than the males, but males are more vulnerable than the females.

## CONFLICT OF INTEREST

The authors declare no competing interest.

### PEER REVIEW

The peer review history for this article is available at https://publons.com/publon/10.1002/brb3.2723

## Data Availability

The data sets used and analyzed in the present research are available from the corresponding author on reasonable request.

## References

[brb32723-bib-0001] Ajayi, A. F. , & Akhigbe, R. E. (2020). Staging of the estrous cycle and induction of estrus in experimental rodents: An update. Fertility Research and Practice, 6(1), 1–15. 32190339 10.1186/s40738-020-00074-3 PMC7071652

[brb32723-bib-0002] Aliheydari, M. , Ghotbeddin, Z. , Khazaeil, K. , & Tabandeh, M. R (2020). Effect of fish oil treatment during chronic hypoxia in pregnancy on memory impairment, brain morphometry changes and oxidative stress in adult male rat offspring. Feyz, 24(2), 170–80.

[brb32723-bib-0003] Artemiadis, A. K. , & Anagnostouli, M. C. (2010). Apoptosis of oligodendrocytes and post‐translational modifications of myelin basic protein in multiple sclerosis: Possible role for the early stages of multiple sclerosis. European Neurology, 63, 65–72. 20068323 10.1159/000272940

[brb32723-bib-0004] Avigan, P. D. , Cammack, K. , & Shapiro, M. L. (2020). Flexible spatial learning requires both the dorsal and ventral hippocampus and their functional interactions with the prefrontal cortex. Hippocampus, 30(7), 733–744. 32077554 10.1002/hipo.23198 PMC7731996

[brb32723-bib-0005] Bannerman, P. G. , Hahn, A. , Ramirez, S. , Morley, M. , Bonnemann, C. , Yu, S. , Zhang, G.‐X. , Rostami, A. , & Pleasure, D. (2005). Motor neuron pathology in experimental autoimmune encephalomyelitis: Studies in THY1‐YFP transgenic mice. Brain, 128, 1877–1886. 15901645 10.1093/brain/awh550

[brb32723-bib-0006] Bauer, C. , Dyrby, T. , Sellebjerg, F. , Madsen, K. , Svolgaard, O. , Blinkenberg, M. , Siebner, H. R. , & Andersen, K. W. (2020). Motor fatigue is associated with asymmetric connectivity properties of the corticospinal tract in multiple sclerosis. NeuroImage: Clinical, 28, 102393. 32916467 10.1016/j.nicl.2020.102393 PMC7490847

[brb32723-bib-0007] Bebo, B. F. , Fyfe‐Johnson, A. , Adlard, K. , Beam, A. G. , Vandenbark, A. A. , & Offner, H. (2001). Low‐dose estrogen therapy ameliorates experimental autoimmune encephalomyelitis in two different inbred mouse strains. Journal of Immunology, 166, 2080–2089. 10.4049/jimmunol.166.3.2080 11160259

[brb32723-bib-0008] Berdugo‐Vega, G. , Arias‐Gil, G. , López‐Fernández, A. , Artegiani, B. , Wasielewska, J. M. , Lee, C. C. , Lippert, M. T. , Kempermann, G. , Takagaki, K. , & Calegari, F. (2020). Increasing neurogenesis refines hippocampal activity rejuvenating navigational learning strategies and contextual memory throughout life. Nature Communication, 11, 135. 10.1038/s41467-019-14026-z PMC6952376 31919362

[brb32723-bib-0009] Blakemore, W. F (1982). Ethidium bromide induced demyelination in the spinal cord of the cat. Neuropathology and Applied Neurobiology, 8, 365–375. 7177337 10.1111/j.1365-2990.1982.tb00305.x

[brb32723-bib-0010] Bosoko, N. , & Langdon, D. (2020). The contribution of perceived memory and information processing deficits on multiple sclerosis cognitive difficulties. Journal of Multiple Sclerosis, 7(3), 001–008.

[brb32723-bib-0011] Chen, D. L. , Engle, J. T. , Griffin, E. A. , Miller, J. P. , Chu, W. , Zhou, D. , & Mach, R. H. (2015). Imaging caspase‐3 activation as a marker of apoptosis‐targeted treatment response in cancer. Molecular Imaging and Biology, 17, 384–393. 25344147 10.1007/s11307-014-0802-8 PMC4874215

[brb32723-bib-0012] Chu, T. , Shields, L. , Zeng, W. , Zhang, Y. , Wang, Y. , Barnes, G. , Shields, C. B. , & Cai, J. (2021). Dynamic glial response and crosstalk in demyelination‐remyelination and neurodegeneration processes. Neural Regeneration Research, 16(7), 1359–1368. 33318418 10.4103/1673-5374.300975 PMC8284258

[brb32723-bib-0013] Cohan, S. L. , Hendin, B. A. , Reder, A. T. , Smoot, K. , Avila, R. , Mendoza, J. P. , & Weinstock‐Guttman, B. (2021). Interferons and multiple sclerosis: Lessons from 25 years of clinical and real‐world experience with intramuscular interferon beta‐1a (Avonex). CNS Drugs, 35(7), 743–767. 34228301 10.1007/s40263-021-00822-z PMC8258741

[brb32723-bib-0014] Constantinescu, C. , Farooqi, N. , Brien, K. , & Gran, B. (2011). Experimental autoimmune encephalomyelitis (EAE) as a model for multiple sclerosis (MS). British Journal of Pharmacology, 164(4), 1079–1106. 21371012 10.1111/j.1476-5381.2011.01302.x PMC3229753

[brb32723-bib-0015] Dai, J. , Lee, B. C. , An, P. , Su, Z. , Qu, R. , Eom, K. H. , & Soh, K. S. (2011). In situ staining of the primo vascular system in the ventricles and subarachnoid space of the brain by trypan blue injection into the lateral ventricle. Neural Regeneration Research, 6(28), 2171–2175.

[brb32723-bib-0016] D'Amelio, M. , Cavallucci, V. , & Cecconi, F. (2010). Neuronal caspase‐3 signaling: Not only cell death. Cell Death and Differentiation, 17, 1104–1114. 19960023 10.1038/cdd.2009.180

[brb32723-bib-0017] De Stefano, N. , Airas, L. , Grigoriadis, N. , Mattle, H. P. , O'Riordan, J. , Oreja‐Guevara, C. , Sellebjerg, F. , Stankoff, B. , Walczak, A. , Wiendl, H. , & Kieseier, B. C. (2014). Clinical relevance of brain volume measures in multiple sclerosis. CNS Drugs, 28(2), 147–156. 24446248 10.1007/s40263-014-0140-z

[brb32723-bib-0018] Dobrynina, L. , Gadzhieva, Z. , Shamtieva, K. , Kremneva, E. , Akhmetzyanov, B. , Kalashnikova, L. , & Krotenkova, M. (2020). Microstructural predictors of cognitive impairment in cerebral small vessel disease and the conditions of their formation. Diagnostics (Basel), 10(9), 720. 10.3390/diagnostics10090720 PMC7554972 32961692

[brb32723-bib-0019] Dunn, S. E. , Gunde, E. , & Lee, H. (2015). Sex‐based differences in multiple sclerosis (MS): Part II: Rising incidence of multiple sclerosis in women and the vulnerability of men to progression of this disease. Emerging and Evolving Topics in Multiple Sclerosis Pathogenesis and Treatments, 26, 57–86. 10.1007/7854_2015_370 25690592

[brb32723-bib-0020] Ghaffari, S. , Hatami Nemati, H. , & Dehghan, G. (2013). Protective effect of short‐term administration of ethanolic saffron extract on improvement of cognitive deficits and decrement of lipid peroxidation induced by ethidium bromide in experimental models of MS. Physiology and Pharmacology, 17(3), 315–27.

[brb32723-bib-0021] Goudarzvand, M. , Choopani, S. , Shams, A. , Javan, M. , Khodaii, Z. , Ghamsari, F. , Naghdi, N. , Piryaei, A. , & Haghparast, A. (2016). Focal injection of ethidium bromide as a simple model to study cognitive deficit and its improvement. Basic and Clinical Neuroscience, 7(1), 63–72. 27303601 PMC4892333

[brb32723-bib-0022] He, J. , Yamada, K. , Nakajima, A. , Kamei, H. , & Nabeshima, T. (2002). Learning and memory in two different reward tasks in a radial arm maze in rats. Behavioural Brain Research, 134(1‐2), 139–148. 12191800 10.1016/s0166-4328(01)00460-0

[brb32723-bib-0023] Horakova, D. , Dwyer, M. G. , Havrdova, E. , Cox, J. L. , Dolezal, O. , Bergsland, N. , Rimes, B. , Seidl, Z. , Vaneckova, M. , & Zivadinov, R. (2009). Gray matter atrophy and disability progression in patients with early relapsing‐remitting multiple sclerosis: A 5‐year longitudinal study. Journal of the Neurological Sciences, 282(1‐2), 112–119. 19168190 10.1016/j.jns.2008.12.005

[brb32723-bib-0024] Huang, K. , Fang, W. , Li, A. , Liang, P. H. , Wu, C. H. , Shyr, Y. , & Yang, M.‐H. (2018). Caspase‐3, a key apoptotic protein, as a prognostic marker in gastric cancer after curative surgery. International Journal of Surgery, 52, 258–263. 29501797 10.1016/j.ijsu.2018.02.055

[brb32723-bib-0025] Jin, J. , Ko, I. , Kim, S. , Shin, M. S. , Kim, S. H. , & Jee, Y. S. (2014). Swimming exercise ameliorates multiple sclerosis‐induced impairment of short‐Term memory by suppressing apoptosis in the hippocampus of rats. Journal of Exercise Rehabilitation, 10(2), 69–74. 24877040 10.12965/jer.140103 PMC4025552

[brb32723-bib-0026] Juliette, B. , Aurélie, R. , Mathilde, D. , Julie, C. , Aurore, S. , Bruno, B. , Tourdias, T. , & Koubiyr, I. (2021). Insights on the relationship between hippocampal connectivity and memory performances at the early stage of multiple sclerosis. Frontiers in Neurology, 12(19), 752. 10.3389/fneur.2021.667531 PMC8170471 34093415

[brb32723-bib-0027] Jurkowski, M. , Bettio, L. , Woo, E. K. , Patten, A. , Yau, S. , & Gil‐Mohapel, J. (2020). Beyond the hippocampus and the SVZ: Adult neurogenesis throughout the brain. Frontiers in Cellular Neuroscience, 14, 576444. 33132848 10.3389/fncel.2020.576444 PMC7550688

[brb32723-bib-0028] Koike, H. , & Katsuno, M. (2021). Macrophages and autoantibodies in demyelinating diseases. Cells, 10(4), 844. 33917929 10.3390/cells10040844 PMC8068327

[brb32723-bib-0029] Libbey, J. E. , & Fujinami, R. S. (2021). Viral mouse models used to study multiple sclerosis: Past and present. Archives of Virology, 166(4), 1015–1033. 33582855 10.1007/s00705-021-04968-5 PMC7882042

[brb32723-bib-0030] Linker, R. A. , & Lee, D. H. (2021). Models of autoimmune demyelination in the central nervous system: On the way to translational medicine. Experimental & Translational Stroke Medicine, 1, 5. (2009). 10.1186/2040-7378-1-5 PMC2816864 20142992

[brb32723-bib-0031] Mazzanti, C. M. , Spanevello, R. , Ahmed, M. , Schmatz, R. , Mazzanti, A. , Salbego, F. Z. , Graça, D. L. , Sallis, E. S. V. , Morsch, V. M. , & Schetinger, M. R. C. (2021). Cyclosporine A inhibits acetylcholinesterase activity in rats experimentally demyelinated with ethidium bromide. International Journal of Developmental Neuroscience, 25(4), 259–264. 10.1016/j.ijdevneu.2007.02.005 17467222

[brb32723-bib-0032] Monaco, S. , Nicholas, R. , Reynolds, R. , & Magliozzi, R. (2020). Intrathecal Inflammation in Progressive Multiple Sclerosis. International Journal of Molecular Sciences, 21(21), 8217. 10.3390/ijms21218217 PMC7663229 33153042

[brb32723-bib-0033] Nickell, C. , Thompson, K. , Pauly, J. , & Nixon, K. (2020). Recovery of hippocampal‐dependent learning despite blunting reactive adult neurogenesis after alcohol dependence. Brain Plasticity, 6(1), 83–101. 33680848 10.3233/BPL-200108 PMC7903006

[brb32723-bib-0034] Paxinos, G. , & Watson, C. (1982). The rat brain in stereotaxic coordinates (6th edn). New York: Academic Press.

[brb32723-bib-0035] Sim, F. J. , Franklin, R. J. M. , & Hinks, G. L. (2000). The re‐expression of the homeodomain transcription factor Gtx during remyelination of experimentally induced demyelinating lesions in young and old rat brain. Neuroscience, 100, 131–139. 10996464 10.1016/s0306-4522(00)00252-9

[brb32723-bib-0036] Sohaili, S. , Moayeri, A. , Darvishi, M. , & Abbasi, N. (2020). Repair Effects of Depakene on Neuropathological Changes in Cuprizone Model of Demyelination. International Journal of Pharmaceutical and Phytopharmacological Research, 10(5), 256–269.

[brb32723-bib-0037] Strik, M. , Cofré Lizama, L. E. , Shanahan, C. , Walt, A. , Boonstra, F. , Glarin, R. , Kilpatrick, T. J. , Geurts, J. J. G. , Cleary, J. O. , Schoonheim, M. M. , Galea, M. P. , & Kolbe, S. C. (2021). Axonal loss in major sensorimotor tracts is associated with impaired motor performance in minimally disabled multiple sclerosis patients. Brain Communications, 3(2), fcab032. 34222866 10.1093/braincomms/fcab032 PMC8244644

[brb32723-bib-0038] Tambalo, S. , Peruzzotti‐Jametti, L. , Rigolio, R. , Fiorini, S. , Bontempi, P. , Mallucci, G. , Balzarotti, B. , Marmiroli, P. , Sbarbati, A. , Cavaletti, G. , Pluchino, S. , & Marzola, P. (2015). Functional magnetic resonance imaging of rats with experimental autoimmune encephalomyelitis reveals brain cortex remodeling. Journal of Neuroscience, 35(27), 10088–10100. 26157006 10.1523/JNEUROSCI.0540-15.2015 PMC4495237

[brb32723-bib-0039] Thornton, A. E. , & Raz, N. (1997). Memory impairment in multiple sclerosis: A quantitative review. Neuropsychology, 11, 357–366. 9223140 10.1037//0894-4105.11.3.357

[brb32723-bib-0040] Ubogu, E. , Yosef, N. , Xia, R. , & Sheikh, K. (2012). Behavioral, electrophysiological, and histopathological characterization of a severe murine chronic demyelinating polyneuritis model. Journal of the Peripheral Nervous System, 17(1), 53–61. 22462666 10.1111/j.1529-8027.2012.00375.x PMC5792059

[brb32723-bib-0041] Vorhees, C. , & Williams, M. (2006). Morris water maze: Procedures for assessing spatial and related forms of learning and memory. Nature Protocols, 1, 848–858. 17406317 10.1038/nprot.2006.116 PMC2895266

[brb32723-bib-0042] Wegner, C. , Esiri, M. M. , Chance, S. A. , Palace, J. , & Matthews, P. M. (2006). Neocortical neuronal, synaptic, and glial loss in multiple sclerosis. Neurology, 67, 960–967. 17000961 10.1212/01.wnl.0000237551.26858.39

[brb32723-bib-0043] Woodruff, R. H. , & Franklin, R. J. M. (1988). The expression of myelin basic protein exon 1 and exon 2 containing transcripts during myelination of the neonatal rat spinal cord—An in‐situ hybridization study. Journal of Neurocytology, 27, 683–693. 10.1023/a:1006972316697 10447242

[brb32723-bib-0044] Yao, Z. , Fu, Y. , Wu, J. , Zhang, W. , Yu, Y. , Zhang, Z. , Wu, X. , Wang, Y. , & Hu, B. (2020). Morphological changes in subregions of hippocampus and amygdala in major depressive disorder patients. Brain Imaging and Behavior, 14(3), 653–667. 30519998 10.1007/s11682-018-0003-1 PMC6551316

[brb32723-bib-0045] Ziehn, M. O. , Avedisian, A. A. , Dervin, S. M. , Umeda, E. A. , O'Dell, T. J. , & Voskuhl, R. R. (2012). Therapeutic testosterone administration preserves excitatory synaptic transmission in the hippocampus during autoimmune demyelinating disease. The Journal of Neuroscience: The Official Journal of the Society for Neuroscience, 32, 12312–12324. 22956822 10.1523/JNEUROSCI.2796-12.2012 PMC3571760

[brb32723-bib-0046] Zivadinov, R. , & Bakshi, R. (2004). Role of MRI in multiple sclerosis. II. Brain and spinal cord atrophy. Frontiers in Bioscience, 9, 647–664. 14766398 10.2741/1262

